# Parent-Reports of Sex-Typed Play Preference in Preschool Children: Relationships to 2D:4D Digit Ratio and Older Siblings’ Sex

**DOI:** 10.1007/s10508-020-01662-6

**Published:** 2020-03-28

**Authors:** Lisa M. Körner, Marie Luisa Schaper, Bettina M. Pause, Martin Heil

**Affiliations:** Department of Experimental Psychology, Institute of Experimental Psychology, Heinrich Heine University Düsseldorf, Universitätsstraße 1, 40225 Düsseldorf, Germany

**Keywords:** Sex differences, 2D:4D, Prenatal testosterone, Sex-typed play preference, Preschool Activities Inventory

## Abstract

Sex-typed play behavior shows large sex differences and seems to be affected by prenatal sex hormones. For example, a smaller, more male-typical ratio between the second and fourth digit length (2D:4D), a proposed marker for prenatal testosterone exposure, has been shown to be related to sex-typed play preference in childhood. Nevertheless, it is still being debated whether 2D:4D displays a stable sex difference throughout childhood, as there are few longitudinal studies. In the present study, children’s 2D:4D was measured on both hands on four occasions from early infancy to early childhood (T1: 5 months, T2: 9 months, T3: 20 months, and T4: 40 months) providing the rare possibility to test the temporal stability of the sex difference. Parents completed the Preschool Activities Inventory at T4 and reported on the number of older brothers and sisters as a measure for socialization influences. Parents described boys as playing more masculine and less feminine than girls. Boys had smaller 2D:4D than girls at all measurements (T1–T4) and on both hands (right/left). Nevertheless, 2D:4D increased significantly from T3 to T4 in both sexes. Girls, but not boys, who were described as playing more masculine and less feminine had more masculine 2D:4D ratios at T1–T4 on both hands (except for right 2D:4D at T2 and T3) and had more older brothers and fewer older sisters. These data underline the stability of the sex difference in 2D:4D and show the importance of both biological and social influences on sex-typed play behavior.

## Introduction

Sex-typed toy preference is one of the earliest observed sex differences in behavior, becoming apparent in children as young as 12 months (Servin, Bohlin, & Berlin, 1999; Todd, Barry, & Thommessen, 2017; Todd et al., 2018; van de Beek, van Goozen, Buitelaar, & Cohen-Kettenis, 2009). Whereas such sex differences in toy preference are only modest in size around one year of age (for a review, see Zosuls & Ruble, 2018), they increase with age (Golombok et al., 2008; for a review, see Todd et al., 2018) with very large effect sizes of about Cohen’s *d* = 3 for preschool and primary school children (for a review and meta-analysis, see Davis & Hines, 2020; Hines, 2010). However, the size of these sex differences depends on the method used to determine sex-typed play preference. Observational studies typically find sex differences for playing with cars/trucks and dolls (for reviews, see Davis & Hines, 2020; Zosuls & Ruble, 2018) that are smaller than for parents’ reports on sex-typed play preferences in a questionnaire (for a review, see Hines, 2010). Noticeably, not only children, but also non-human primates seem to prefer to play with sex-specific toys (Alexander & Hines, 2002; Hassett, Siebert, & Wallen, 2008). This is notable because non-human primates are obviously less subject to social influences. Both the early appearance of sex-specific play preferences in children, as well as the evidence from non-human primates, suggest a biological component in the emergence of sex-typed play preferences in addition to socialization influences.

Sex hormones that are involved in the development of primary and secondary sexual characteristics in ontogenesis are a likely candidate for such a biological component. However, sex-typed play behavior appears long before puberty in a time frame in which there are no differences in sex hormone levels between boys and girls (Hines, 2010). Following from this, early prenatal effects of sex hormones have been suggested to impact sex-typed preferences in children (for a review, see Berenbaum & Beltz, 2016). For example, girls with congenital adrenal hyperplasia (CAH) who are exposed to high levels of androgens prenatally and in the early postnatal period show more male-typical play behavior than unaffected controls (Berenbaum & Hines, 1992; Nordenström, Servin, Bohlin, Larsson, & Wedell, 2002; Pasterski et al., 2005) and parents describe their daughters behavior as more masculine in comparison with unaffected female relatives (Hines, 2003). Additionally, women with CAH retrospectively describe their play behavior as more masculine (Hines, Brook, & Conway, 2004). This is further corroborated by experimental studies on female rhesus macaques that displayed a strong increase in rough-and-tumble play after they had been prenatally treated with androgens (for a review, see Thornton, Zehr, & Loose, 2009).

Notably, testosterone levels differ between boys and girls from around Week 8 of gestation (Judd, Robinson, Young, & Jones, 1976) and are presumed to have organizational effects on the brain. In turn, they should affect sex differences in behavior later in life (for a review, see Cohen-Bendahan, van de Beek, & Berenbaum, 2005). In this context, one study indicated that girls and boys exposed to higher levels of testosterone in amniotic fluid showed more male-typical play behavior later in life (Auyeung et al., 2009). By contrast, two other amniocentesis studies were not able to find this relationship (Knickmeyer et al., 2005; van de Beek et al., 2009). However, all three studies used different measures to quantify sex-typed play preferences and the children differed in age.

Thus, amniocentesis studies, which are very rare due to the high effort involved, show inconsistent results. However, studies on the relationship between sex-typed play behavior and the ratio between the second and fourth digit length (a presumed marker for the prenatal testosterone level) show more consistent results (Hönekopp & Thierfelder, 2009; Mitsui et al., 2016; Wong & Hines, 2016). Following the observation that females have larger ratios between the second and fourth digit (2D:4D) than males, Manning, Scutt, Wilson, and Lewis-Jones (1998) suggested the 2D:4D as an easily accessible marker for prenatal testosterone exposure.

Two meta-analyses confirmed a sex difference in 2D:4D (Grimbos, Dawood, Burriss, Zucker, & Puts, 2010; Hönekopp & Watson, 2010). This sex difference is already established prenatally as suggested by two studies on aborted fetuses (Galis, Ten Broek, Van Dongen, & Wijnaendts, 2010; Malas, Dogan, Hilal Evcil, & Desdicioglu, 2006). In favor of 2D:4D as a marker for prenatal testosterone exposure, females with CAH tend to have masculinized (i.e., smaller) 2D:4D (Brown, Hines, Fane, & Breedlove, 2002; Ökten, Kalyoncu, & Yaris, 2002). In mice, increasing androgens and reducing estrogens in utero decreased 2D:4D (Zheng & Cohn, 2011).

Even though there is convincing evidence that 2D:4D is influenced prenatally by sex hormones, the specific relationship with prenatal sex hormone levels from amniotic fluid and umbilical cord blood in humans is still not fully understood. There are only two published studies that link 2D:4D to prenatal sex hormones from amniotic fluid with inconsistent findings. One study found that more masculine 2D:4D on the right hands of 29 2-year-olds (not separated for sex) were associated with a higher ratio of testosterone to estradiol levels (Lutchmaya, Baron-Cohen, Raggatt, Knickmeyer, & Manning, 2004), while another study found that the right and left 2D:4D in newborn girls (but not boys) to be correlated with amniotic fluid testosterone (Ventura, Gomes, Pita, Neto, & Taylor, 2013). Only one study using umbilical cord blood (sampled at birth) to measure sex hormones found an expected negative relationship between testosterone and left 2D:4D in girls (Whitehouse et al., 2015), while others failed to show this negative relationship (Çetin, Can, & Özcan, 2016; Hickey et al., 2010; Hollier et al., 2015). Additionally, in case testosterone effects on 2D:4D are mediated by androgen receptor type, there should be a positive correlation between functional androgen receptor gene variation (CAG stretches) and 2D:4D. Two meta-analyses have failed to show a relationship between 2D:4D and CAG stretches (Hönekopp, 2013; Voracek, 2014). Therefore, 2D:4D as a marker for prenatal hormone exposure should be interpreted with caution (for an overview of existing evidence, see Richards, 2017).

Assuming that 2D:4D is determined (at least partially) by prenatal hormones, the temporal stability of the sex difference in 2D:4D should be high, and sex differences should be present early in life. While a cross-sectional study on 2- to 25-year-old individuals found no age differences in 2D:4D (Manning et al., 1998), another cross-sectional study showed an increase in 2D:4D with age in 2- to 5-year-olds (Williams, Greenhalgh, & Manning, 2003). Rare longitudinal studies, however, are better suited to evaluate the stability of 2D:4D controlling for inter-individual differences. Such studies have shown a slight increase in 2D:4D with age (McIntyre, Cohn, & Ellison, 2006: T1: 6–7 years, T2: 8–9 years; McIntyre, Ellison, Lieberman, Demerath, & Towne, 2005: T1: 1 year, T2: 5 years, T3: 9 years, T4: 13 years, T5: 17 years; Trivers, Manning, & Jacobson, 2006: T1: 7–13 years, T2: 11–17 years; Wong & Hines, 2016: T1: 20-40 months, T2: 26-47 months). However, high correlations between the measurements (Pearson’s *r* = .71–.88) suggest a high temporal stability of 2D:4D. By contrast, one longitudinal study on 0- to 2-year-olds showed a decrease of 2D:4D in the first year and an increase in the second year of life and low correlations (Pearson’s *r* = .35–.53) between measurements (Knickmeyer, Woolson, Hamer, Konneker, & Gilmore, 2011: T1: 2 weeks, T2: 12 months, T3: 24 months).

Supporting the validity of 2D:4D, three studies (Hönekopp & Thierfelder, 2009; Mitsui et al., 2016; Wong & Hines, 2016) have shown that children with lower 2D:4D (and, thus, supposedly higher testosterone exposure in utero) display more masculine play behavior (as described by the parents’ answers on the Preschool Activities Inventory; PSAI; Golombok & Rust, 1993). Nevertheless, there is considerable discrepancy concerning the side of the hand (right/left) and the sex of the children in which the correlations were found (Hönekopp & Thierfelder, 2009: left 2D:4D of boys; Mitsui et al., 2016: right and left 2D:4D of boys; Wong & Hines, 2016: right 2D:4D of boys and right and left 2D:4D of girls).

In addition to a likely biological effect on play behavior, socialization plays an important role. It is well known that children’s (sex-specific) toy preferences are influenced by parents, teachers, (older) siblings, and peers. For example, children are often reinforced for sex-congruent behavior (for reviews, see Berenbaum, Blakemore, & Beltz, 2011; Hines, 2010). Whereas the reinforcement of sex-congruent behavior of parents and teachers and the influence of peers is difficult to quantify, studies that have recorded the number of older brothers and sisters have shown that both girls and boys with more older brothers displayed more male-typical and less female-typical behavior and with more older sisters more female-typical and less male-typical play behavior (Hines et al., 2002b; Mitsui et al., 2016; Rust et al., 2000). According to social cognitive theory, this effect of older siblings is explained by observational learning and thus as a socialization factor (Berenbaum et al., 2011; Rust et al., 2000), which seems plausible because children may play with older siblings and their toys. However, it cannot be ruled out that the influence of older siblings on the behavior of younger siblings may also (partly) be based on a genetic or hormonal and, thus, a biological component (Berenbaum et al., 2011). In this context, the fraternal birth-order effect is worth mentioning as it describes the increased probability for a younger brother to be gay with an increasing number of older brothers. This effect is explained as a consequence of a progressive immunization of some mothers against male antigens with each pregnancy with a male fetus and a simultaneous increase in antibodies that affect the sexual differentiation of the brain (for a meta-analysis, see Blanchard, 2018). Bogaert et al. (2018) found antibody levels against the Y-linked protein NLGN4Y that is important in brain development, to be higher in mothers of gay sons than in the control samples. Additionally, in a large community sample, later fraternal birth order was related to elevated gender variance in boys (Coome, Skorska, van der Miesen, Peragine, & VanderLaan, 2018) which indicates that the progressive immunization hypothesis is not only valid for homosexuality but also for sex-typed behavior in childhood. However, this effect has only been shown for boys with older brothers, so that the observed relationship between the sex-typed play behavior of boys and the number of older sisters as well as the relationship between the sex-typed play behavior of girls and the number of older brothers and sisters (Hines et al., 2002b; Mitsui et al., 2016; Rust et al., 2000) cannot be attributed to the birth-order effect and therefore most likely indicates socializing effects. To date, there are no studies that investigated both the influence of older siblings and prenatal hormonal effects in order to be able to make a statement as to whether the effects are additive or interactive (Berenbaum et al., 2011).

The current study aims at clarifying the relationship between sex-typed play behavior and 2D:4D. Only one of the three studies examining this relationship (Hönekopp & Thierfelder, 2009; Mitsui et al., 2016; Wong & Hines, 2016) assessed digit ratio in a longitudinal design (with only two measurements on 2- to 3-year-olds over a 6- to 8-month period; Wong & Hines, 2016). By contrast, the present longitudinal study consisted of four measurements of the digit ratios from both hands at different ages starting in early infancy (T1: 5 months, T2: 9 months, T3: 20 months, and T4: 40 months). The sex differences in 2D:4D should be stable if they are indeed influenced by prenatal testosterone. Therefore, we need multiple measurements at different ages during early infancy and childhood to draw conclusions about 2D:4D as a potential marker for prenatal testosterone. Our longitudinal design has a clear advantage over a cross-sectional design because it reduces inter-individual variance and increases the signal-to-noise ratio. At T4, parents completed the PSAI (Golombok & Rust, 1993) to record sex-typed play behavior of their children. The PSAI is a standardized, frequently used measure for play preferences that shows large sex differences (Hönekopp & Thierfelder, 2009; Mitsui et al., 2016; Wong & Hines, 2016) which are typically larger than in direct observations of toy preferences in a single, unnatural laboratory situation (Hines, 2010; Wong & Hines, 2016). It also has the advantage that it not only asks for toy preferences, but also for activity preferences and temperamental characteristics during the last month, giving a more comprehensive picture of sex-typed play behavior in comparison with observational studies of toy preferences.

Additionally, the number of older brothers and sisters (living in the same household) was assessed as an indicator for socialization effects on sex-typed play behavior (Mitsui et al., 2016; Rust et al., 2000). This allows us, for the first time, to assess the combination of potential biological (2D:4D) and socialization influences (older siblings) on sex-typed play behavior (Berenbaum et al., 2011).

Based on previous research, we predicted more male-typical play behavior in boys than in girls (operationalized by the PSAI score) and lower digit ratios in boys than in girls, independent of age and hand (right/left). For the temporal stability of 2D:4D, we expected a slight increase with age independent of the sex difference. Moreover, we predicted that boys and girls with lower digit ratios (supposedly higher prenatal testosterone exposure) should display more male-typical and less female-typical play behavior (higher PSAI scores). With respect to older siblings, more older brothers should lead to more masculine and less feminine play behavior (higher PSAI scores) and more older sisters to less masculine and more feminine behavior (lower PSAI scores) in both boys and girls.

## Method

### Participants

Between 2010 and 2012, *Praenatal.de* (Düsseldorf, Germany) recruited mothers who underwent prenatal screening. A total of 388 mothers agreed to be contacted by the Department of Experimental Psychology of the Heinrich Heine University Düsseldorf after birth of their children. The prerequisites for participation in the study were no identified prenatal abnormalities of the child and an uncomplicated course of pregnancy. We did not contact mothers that had significant problems later during pregnancy or childbirth. All remaining 274 mothers whose children had an APGAR score (Apgar, 1953) of at least nine were contacted for the study. All children were Caucasian and were born between January 2011 and February 2013 in and around Düsseldorf.

Children were tested on four occasions, at the age of 5 months (T1), 9 months (T2), 20 months (T3), and 40 months (T4; for the number of participants and mean ages at all four time points, see Table 1) in the Department of Experimental Psychology at the University of Düsseldorf. For 78 boys and 75 girls, parents’ answers on the Preschool Activities Inventory (PSAI; Golombok & Rust, 1993) were assessed at T4.

**Table 1:** Number and age (in months) of participants at T1–T4

Time point			Boys	Girls	Total
T1: 5 months		*N* *M* SD	114 5.40 0.29	111 5.45 0.31	225 5.43 0.30
T2: 9 months		*N* *M* SD	101 9.38 0.35	91 9.36 0.39	192 9.37 0.37
T3: 20 months		*N* *M* SD	86 20.54 0.34	80 20.52 0.44	166 20.53 0.39
T4: 40 months		*N* *M* SD	80 40.49 0.51	78 40.55 0.65	158 40.52 0.59

At each measurement, parents gave informed consent for participation, recording, and storage of data and received a refund of their travel expenses. The study was approved by the local Ethics Committee of the Science Faculty of the University of Düsseldorf, Germany.

## Materials and Procedure

### Preschool Activities Inventory

The German version of the standardized questionnaire Preschool Activities Inventory (PSAI; Golombok & Rust, 1993) by Hönekopp and Thierfelder (2009) was used to measure the children’s sex-typed play behavior. The questionnaire was presented on a laptop screen with the software *Presentation* (Neurobehavioral Systems, USA) and completed by the accompanying parent (116 mothers, 9 fathers, 28 both parents) at T4 (age: 40 months). The PSAI consists of 24 items assessing children’s toy preferences (7 items), activity preferences (11 items), and temperamental characteristics (6 items). Twelve items assess typical masculine and 12 typical feminine behavior on a 5-point scale. The PSAI is scored by subtracting the sum of the “female items” from the sum of “the male items,” and transforming the score into a pseudo-*T* scale (Golombok & Rust, 1993). Thus, a higher score indicates more masculine behavior and a lower score more feminine behavior.

#### 2D:4D

As hand scans (or photocopies) produce both larger sex differences (Hönekopp & Watson, 2010) and a higher measurement precision (Kemper & Schwerdtfeger, 2009; Mikac, Buško, Sommer, & Hildebrandt, 2016) than direct measurements (with calipers), both hands of the children were scanned at T1, T2, T3, and T4, respectively. The freeware program *Autometric* (DeBruine, 2004) was used to determine the ratio between the second and the fourth digit (2D:4D, the midpoint of the ventral proximal crease of the second digit to the tip of the second digit, and the ventral proximal crease of the fourth digit to the tip of the fourth digit). *Autometric* has been specially designed to measure finger length ratios and has been shown to be the superior program for indirect 2D:4D measurement compared to other computer-based measurement methods because of its high reliability (Kemper & Schwerdtfeger, 2009; Mikac et al., 2016). Scans in which the tips or the ventral creases could not be identified were excluded. One rater measured all hand scans (max. 8 per child) twice, and a second independent rater measured all hand scans once. The raters were blind to the sex of the children. The intra- and inter-rater reliabilities were determined with intra-class correlations (all ICC > .90; see Table 2). The three measurements were averaged to increase reliability. Due to dropout of participants in the course of the study and exclusion of non-measurable hand scans, the number of available hand scans per child varied between one and eight scans. Since it is more difficult to scan the hands of babies and infants as they cannot be instructed to hold still and place the hand flat on the scanner, there are less measurable hand scans at T1 to T3 compared to T4 (measurable hand scans: T1: 64.9%, T2: 60.7%, T3: 60.8%, T4: 78.5%).

**Table 2:** Intra- and inter-rater reliabilities (ICCs) for 2D:4D

	5 mos. Right hand	5 mos. Left hand	9 mos. Right hand	9 mos. Left hand	20 mos. Right hand	20 mos. Left hand	40 mos. Right hand	40 mos. Left hand
Intra-rater	.97	.96	.96	.95	.92	.96	.96	.96
Inter-rater	.94	.94	.96	.95	.94	.92	.95	.96

*mo.* months, *Intra-rater* intra-class correlations for two measurements of first rater; *Inter-rater* intra-class correlations for mean of two measurements of first rater and measurement of second rater

### Statistical Analyses

To test whether age, sex, and hand affected 2D:4D, we calculated a multilevel linear regression model (cf. Kenny, Korchmaros, & Bolger, 2003; Krull & MacKinnon, 2001) using the R packages lme4 and lmerTest (Bates, Mächler, Bolker, & Walker, 2014; Kuznetsova, Brockhoff, & Christensen, 2014; R Core Team, 2019), with participants as random effects. As such, the analysis accounts for participant heterogeneity as well as missing data. We entered age (months since birth, *z*-standardized), sex (dummy-coded as boys = 0, girls = 1), and hand (dummy-coded as left = 0, right = 1) as predictors of 2D:4D (*z*-standardized). Thus, the intercept is the average *z*-standardized 2D:4D for left-handed boys (with *z*-standardized age = 0). To follow-up on the effect of age on 2D:4D, three additional multilevel linear regression models comparing the effect of age between T1 and T2, T2 and T3, and T3 and T4 were calculated. Mixed models were also used in the longitudinal study of 2D:4D by Knickmeyer et al. (2011).

The sex difference in sex-typed play behavior (PSAI) was evaluated using an independent sample *t* test. Pearson correlations were calculated to examine the relationships between PSAI and 2D:4D in both sexes separately for every time point and both hands. Correlations were tested for significant differences with Fisher *z*-transformations. Finally, multiple regression analyses (forced entry) were conducted to predict sex-specific play preference from the predictors 2D:4D (average of left and right hand) at T4 (age: 40 months), older brothers and older sisters, separately for girls and boys.

Significance levels were set to .05 for all comparisons. Effect sizes (Cohen’s *d* and Pearson *r*) were interpreted according to Cohen (1988)—small effect *d* ≥ 0.20 or *r* ≥ .10, medium effect *d* ≥ 0.50 or *r* ≥ .30, and large effect *d* ≥ 0.80 or *r* ≥ .50.

## Results

**Fig. 1:**
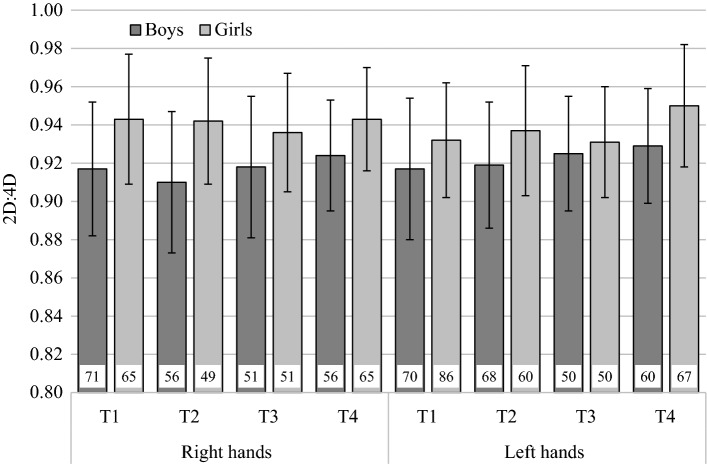
2D:4D at all ages (T1 = 5 months, T2 = 9 months, T3 = 20 months, T4 = 40 months) for both hands, separately for boys and girls. The error bars depict standard deviations. The number in the bars indicates the sample sizes

The multilevel linear regression model indicated a significant main effect of sex. As expected, girls had larger 2D:4D ratios than boys (see Fig. 1). The regression weight indicated that this effect was in the order of 0.49 standard deviations. Further, there was a significant main effect of age with a standardized regression weight of 0.11. Older participants had larger 2D:4D. There was no main effect of hand (see Table 3). Three additional multilevel linear regression models compared the effect of age between T1 and T2, T2 and T3, and T3 and T4 (for the standardized regression weights, see Table 4). These models showed that age did not affect 2D:4D between T1 and T2, and between T2 and T3. However, there was a significant age effect between T3 and T4, indicating that older participants had larger 2D:4D (for descriptive statistics, see Table 5).

**Table 3:** Multilevel linear model results regarding effects of sex, age, and hand on 2D:4D

Effect	Estimate	SE	*df*	*t*	*p*
Intercept	− 0.22	0.07	283.2	3.21	.001
Sex	0.49	0.09	211.1	5.42	< .001
Age	0.11	0.03	848.0	4.17	< .001
Hand	− 0.04	0.05	782.8	0.74	.458

*SE* standard error

Estimates represent standardized (age) and semi-standardized (sex, hand) regression weights. Analyses were performed with the R procedures lme4 and lmerTest with restricted maximum likelihood estimation. For sex, 0 = boys, 1 = girls. For hand, 0 = left, 1 = right

**Table 4:** Multilevel linear model results regarding the age effect on 2D:4D

Measurement	Estimate	SE	*df*	*t*	*p*
T1 vs. T2	− 0.02	0.04	437.6	0.56	.579
T2 vs. T3	0.03	0.04	379.7	0.56	.574
T3 vs. T4	0.16	0.04	375.8	3.88	< .001

*SE* standard error

Estimates represent standardized regression weights. Analyses were performed with the R procedures lme4 and lmerTest with restricted maximum likelihood estimation. For sex, 0 = boys, 1 = girls. For hand, 0 = left, 1 = right. For age, T1 = 5 months (4.6–6.9 months), T2 = 9 months (7.6–10.3 months), T3 = 20 months (18.6–21.7 months), T4 = 40 months (38.3–42.6 months)

**Table 5:** Pearson correlations between PSAI and right and left 2D:4D at T1–T4 and Fisher’s *z*-tests

	Boys			Girls			Fisher’s *z*-test	
	*r*	*p*	*N*	*r*	*p*	*N*	*z*	*p*
T1: Right hand	.08	.308	46	− .39	.005**	43	2.24	.013*
T1: Left hand	− .01	.481	47	− .34	.005**	56	1.69	.046*
T2: Right hand	.13	.225	37	.02	.448	37	0.46	.324
T2: Left hand	.14	.175	45	− .29	.029*	45	2.01	.022*
T3: Right hand	− .05	.382	36	− .21	.093	43	0.69	.244
T3: Left hand	.15	.190	38	− .43	.002**	43	2.64	.004**
T4: Right hand	− .20	.069	55	− .34	.003**	63	0.80	.212
T4: Left hand	− .20	.063	58	− .27	.017*	64	0.40	.345

T1: age 5 months, T2: age 9 months, T3: age 20 months, T4: age 40 months

*p < .05, one-tailed; **p < .01, one-tailed

As expected, PSAI scores for boys (*M* = 64.99, SD = 9.22) were significantly higher than for girls (girls: *M* = 36.03, SD = 10.07), indicating more masculine and less feminine behavior, *t*(151) = 18.52, *p* < .001, *d* = 3.00. Since the parents completed the PSAI at T4, we do not have measurements of the sex-typed play preference for children that did not take part at T4. Consequently, the sample sizes for the correlational analyses of the relationship between 2D:4D and PSAI scores differ depending on the number of measureable hand scans and participation in T4 (for the results of the correlational analyses of every single measurement and Fisher’s *z*-test for differences in correlations between girls and boys, see Table 5). In accordance with the hypothesis, girls with lower 2D:4D showed more masculine and less feminine behavior (higher PSAI scores). This correlation was shown for every time point and both hands except from right 2D:4D at T2 and T3. There were no significant correlations between 2D:4D and PSAI scores in boys.

Boys and girls did not differ in the number of older brothers, *t*(150) = 0.26, *p* = .792, or sister, *t*(126.85) = 1.56, *p* = .122, unequal variances. Correlations between the PSAI score and the number of older brothers and the number of older sisters revealed that, in girls, having more older brothers resulted in higher PSAI scores (more masculine and less feminine behavior; *r*(75) = .42, *p* < .001) and having more older sisters resulted in lower PSAI scores (less masculine and more feminine behavior; *r*(75) = − .28, *p* = .014). There were no significant correlations for boys (older brothers: *r*(77) = .17, *p* = .137; older sisters: *r*(77) = − .07, *p* = .528).

The regression analysis for girls identified a lower 2D:4D at T4 and a higher number of older brothers as significant predictors of more masculine-typed and less feminine-typed play behavior (higher PSAI scores; see Table 6). Partial correlations revealed that both predictors explained independent proportions of variance of the sex-specific play preference in girls (see Table 6). In boys, neither 2D:4D nor the number of older brothers or the number of older sisters significantly explained the PSAI scores (see Table 6).

**Table 6:** Linear model of predictors (2D:4D at T4, older brothers and older sisters) of sex-typed play preference (PSAI scores)

Sex	*R*^*2*^	Predictors	*b*	SE *b*	*β*	*p*	Partial correlation
Boys	.12	2D:4D	− 66.60	36.02	− .22	.069	− .23
		Brothers	3.14	1.67	.22	.064	.23
		Sisters	− 2.36	2.17	− .13	.282	− .13
Girls	.30	2D:4D	− 117.09	42.75	− .29	.008	− .32*
		Brothers	5.02	1.53	.36	.002	− .38*
		Sisters	− 2.83	1.67	− .18	.095	− .21

2D:4D = averaged right and left digit ratios at T4 (age: 40 months); Brothers = number of older brothers, Sisters = number of older sisters

*p < .05

## Discussion

Our study aimed at clarifying the relationship between 2D:4D digit ratio as a putative marker for prenatal testosterone exposure and sex-typed play behavior. Additionally, we explored the effect of the number of older brothers and sisters as markers for the impact of socialization on sex-typed play behavior. As expected, a smaller 2D:4D was associated with more male-typical and less female-typical play behavior (higher PSAI scores), replicating previous studies (Hönekopp & Thierfelder, 2009; Mitsui et al., 2016; Wong & Hines, 2016). However, these expected correlations were only found for girls for every measurement and both hands except for right 2D:4D at T2 (age: 9 months) and T3 (age: 20 months). In addition to the relationship between digit ratio and play behavior, in the present sample, girls were reported to be more masculine and less feminine the more older brothers and less masculine and more feminine the more older sisters they had. These results match those observed in earlier studies (Mitsui et al., 2016; Rust et al., 2000), albeit in contrast to these studies, we found no significant correlations for boys. Additionally, in girls, 2D:4D at T4 and older brothers explained independent proportions of variance of sex-typed play behavior.

The now repeatedly shown relationship between sex-typed play behavior and digit ratios further encourages the assumptions that (1) 2D:4D could be a marker for prenatal sex hormone exposure and (2) sex-typed play preference could be partly affected by these hormones that have organizational effects on the brain. However, we cannot infer from the available data whether 2D:4D is indeed influenced by prenatal sex hormones. Interestingly, in the female sample, six of eight correlations between 2D:4D (four time points, right and left hand) and sex-typed play preference (PSAI at T4) were significant, while Wong and Hines (2016) demonstrated significant correlations only for their older sample (mean age of the girls: *M* = 36.10 months, SD = 5.78 months). This difference might be due to the fact that Wong and Hines (2016) administered the PSAI, a questionnaire designed to assess the behavior of preschoolers, at both time points even though the children were strictly speaking not preschoolers at the time of the first measurement (mean age of the girls: *M* = 29.22 months, SD = 5.51 months).

In contrast to previous research (Hönekopp & Thierfelder, 2009; Mitsui et al., 2016; Wong & Hines, 2016), the expected correlations were only found for girls. One possible explanation could be a threshold effect with prenatal sex hormones affecting 2D:4D and/or play behavior to a specific point with higher levels having no further effect (Breedlove, 2010; Cohen-Bendahan et al., 2005). The fact that boys are prenatally exposed to much higher levels of testosterone than girls might explain the nonsignificant correlations for boys (Hines et al., 2002a). This hypothesis fits with studies on children affected by CAH. Many studies have shown behavioral differences between CAH girls and unaffected female controls but not between CAH boys and unaffected male controls (Berenbaum et al., 2011). Likewise, the only study that has found a significant negative relationship between amniotic testosterone levels and 2D:4D only found it for girls. It was argued that male fetuses are exposed to such high levels of testosterone that levels above average do not impact the 2D:4D ratio any further (Ventura et al., 2013). The only amniocentesis study that has found the expected positive correlation between male-typed play behavior (PSAI) and prenatal amniotic fluid testosterone has also shown a significantly stronger correlation for girls (*r* = .42) than for boys (*r* = .20; Fisher’s *z* = 1.76, *p* = .04; Auyeung et al., 2009). Another explanation could be the different socialization of boys and girls. Boys are generally more often reinforced for sex-typical behavior and more likely to be discouraged from sex-untypical behavior than girls (Fagot, 1978; Langlois & Downs, 1980; Pasterski et al., 2005). Accordingly, previous studies showed that, although the sex difference in play behavior persists throughout childhood, the preference of boys for male-typed play behavior increases with age, while that of girls for female-typed play behavior decreases in favor of an increasing preference for typically masculine toys (Servin et al., 1999; Todd et al., 2017). For this reason, a potential effect of prenatal sex hormones on play behavior in boys could have been masked by a stronger social impact on boys play behavior as compared to girls (Hines et al., 2002a, 2002b).

In addition to the presumed biological effect on sex-typed play preference, reflected in the shown correlations between 2D:4D and play preference (at least for girls), socialization seems to affect sex differences in behavior to an even greater extent (Berenbaum & Beltz, 2016; Berenbaum et al., 2011). In line with this, girls were more masculine and less feminine the more older brothers and less masculine and more feminine the more older sisters they had. It is worth highlighting that, at least for girls, our data provide some evidence that biology (digit ratio) and socialization (impact of older brothers) serve as independent predictors for the amount of sex-specific play behavior (Berenbaum et al., 2011). Nevertheless, it cannot be ruled out that the effect of older siblings on their younger siblings’ play behavior is not only due to socializing but also biological. In this context, it is known that the probability for younger brothers to be gay as well as to show more gender variance in childhood increases with the number of older brothers (for a meta-analysis, see Blanchard, 2018; Coome et al., 2018). However, in the present study, the effect of older siblings on sex-typed play behavior could only be shown for girls for whom no such birth-order effect is known. Conversely, it is obvious that by growing up in the same household with older siblings, observational learning takes place which most likely affects the behavior of the younger siblings (Mitsui et al., 2016; Rust et al., 2000).

Besides the behavioral correlations, the present study, being the first longitudinal study with four measurements over infancy and early childhood, sheds light on the temporal stability of 2D:4D. The digit ratio has to be sexual dimorphic early in life to present a marker for prenatal testosterone exposure (Manning et al., 1998). The present study revealed a medium-sized sex difference in 2D:4D with higher values for girls than boys comparable to previous studies (for a meta-analysis, see Hönekopp & Watson, 2010). The sex difference in 2D:4D was present at all measurements, confirming previous research suggesting 2D:4D to be a reliable measure (McIntyre et al., 2005, 2006; Trivers et al., 2006; Wong & Hines, 2016). Nevertheless, in line with previous research, 2D:4D increased significantly in size (Knickmeyer et al., 2011; McIntyre et al., 2005; Trivers et al., 2006; Williams et al., 2003; Wong & Hines, 2016) from T3 (age: 20 months) to T4 (age: 40 months).

### Conclusion

In our study, we set out to evaluate the relationship between sex-typed play behavior and digit ratios as a proposed marker for prenatal sex hormone exposure. In line with previous research (Mitsui et al., 2016; Wong & Hines, 2016), we found that girls with lower 2D:4D were described as behaving more masculine and less feminine, suggesting a biological component in the development of sex-related play preference. This biological effect acts independent of socialization from older brothers on the girls’ play behavior. A key strength of the present study was its longitudinal design with four measurements throughout infancy and early childhood which demonstrated the stability of the sex difference in 2D:4D.
